# Rapid Atrial Pacing Promotes Atrial Fibrillation Substrate in Unanesthetized Instrumented Rats

**DOI:** 10.3389/fphys.2019.01218

**Published:** 2019-09-20

**Authors:** Wesam Mulla, Barak Hajaj, Sigal Elyagon, Michal Mor, Roni Gillis, Michael Murninkas, Hadar Klapper-Goldstein, Inbar Plaschkes, Vered Chalifa-Caspi, Sharon Etzion, Yoram Etzion

**Affiliations:** ^1^Cardiac Arrhythmia Research Laboratory, Department of Physiology and Cell Biology, Faculty of Health Sciences, Ben-Gurion University of the Negev, Beer-Sheva, Israel; ^2^Regenerative Medicine and Stem Cell Research Center, Ben-Gurion University of the Negev, Beer-Sheva, Israel; ^3^Shraga Segal Department of Microbiology, Immunology and Genetics, Faculty of Health Sciences Ben-Gurion University of the Negev, Beer-Sheva, Israel; ^4^National Institute for Biotechnology in the Negev, Ben-Gurion University of the Negev, Beer-Sheva, Israel

**Keywords:** atrial remodeling, atrial fibrillation substrate, atrial tachypacing, atrial tachycardia, rodent electrophysiology

## Abstract

**Aim:**

The self-perpetuating nature of atrial fibrillation (AF) has been a subject of intense research in large mammalian models exposed to rapid atrial pacing (RAP). Recently, rodents are increasingly used to gain insight into the pathophysiology of AF. However, little is known regarding the effects of RAP on the atria of rats and mice. Using an implantable device for electrophysiological studies in rodents, we examined on a daily basis, the effects of continuous RAP on the developed AF substrate of unanesthetized rats and mice.

**Methods and Results:**

Aggressive burst pacing did not induce AF at baseline in the large majority of rodents, but repeatedly induced AF episodes in rats exposed to RAP for more than 2 days. A microarray study of left atrial tissue from rats exposed to RAP for 2 days vs. control pacing identified 304 differentially expressed genes. Enrichment analysis and comparison with a dataset of atrial tissue from AF patients revealed indications of increased carbohydrate metabolism and changes in pathways that are thought to play critical roles in human AF, including TGF-beta and IL-6 signaling. Among 19 commonly affected genes in comparison with human AF, downregulation of FOXP1 and upregulation of the KCNK2 gene encoding the Kir2.1 potassium channel were conspicuous findings, suggesting NFAT activation. Further results included reduced expression of MIR-26 and MIR-101, which is in line with NFAT activation.

**Conclusion:**

Our results demonstrate electrophysiological evidence for AF promoting effects of RAP in rats and several molecular similarities between the effects of RAP in large and small mammalian models.

## Introduction

Atrial fibrillation (AF), the most prevalent cardiac arrhythmia in clinical practice, is a growing epidemic and a major cause of stroke, heart failure progression and death (Heeringa et al., 2006; Miyasaka et al., 2006; Chugh et al., 2013; Lau et al., 2017). AF is a progressive arrhythmia with pathophysiology that is complex and multi-factorial in nature (Dobrev et al., 2012; Heijman et al., 2014). Present invasive and pharmacological therapeutic modalities for AF have limited efficacy and significant adverse effects (Woods and Olgin, 2014). These limitations have inspired substantial efforts to improve our understanding of the mechanisms underlying AF, with the premise that improved mechanistic insights will lead to better therapeutic strategies (Dobrev et al., 2012; Heijman et al., 2015).

An important aspect in the pathophysiology of AF is its self-perpetuating nature which has been linked to a remodeling process defined as “atrial tachycardia remodeling” (ATR) resulting from increased activation rate of the atrial myocytes (Wijffels et al., 1995; Nattel, 1999; Members et al., 2006). Studies in large animal models have elucidated that rapid atrial pacing (RAP) and consequent cellular calcium overload within atrial cardiomyocytes, lead to multiple secondary changes in the atrial tissue (Dobrev et al., 2012; Heijman et al., 2014). Downregulation of the Ca^2+^ current mediated by L-type calcium channels and upregulation of Ik1 current mediated by Kir2.1 channels are among the most important electrophysiological findings in this setting (Wakili et al., 2011). Both phenomena can reduce the atrial effective refractory period (AERP), and thereby promote multiple circuit reentry in the atrial tissue (Nattel et al., 2008). Additional factors including reactive oxygen species, activation of Ca^2+^/calmodulin-dependent kinase II (CaMKII), and activation of calcineurin–nuclear factor of activated T lymphocyte (NFAT) signaling have also been implicated (Wakili et al., 2011; Dobrev et al., 2012). In structural terms, histological features of ATR include myolysis, glycogen accumulation, cellular hypertrophy, dedifferentiation and neurogenesis (Chang et al., 2001; Thijssen et al., 2001). AF also increases the levels of atrial fibrosis and TGF-β appears to have a central role in this process (Everett and Olgin, 2007; Dobrev et al., 2012).

Animal models are important for better understanding of the pathophysiology of AF, as well as for evaluation of new drug targets (Nattel et al., 2005). In recent years there is a rapidly increasing interest in the use of rodents to study various aspects in the pathophysiology of AF (Beharier et al., 2007; Kirchhof et al., 2011; Riley et al., 2012; Shan et al., 2012; Iwasaki et al., 2014; Hohl et al., 2017; Skibsbye et al., 2018). Nevertheless, the small and delicate rodent atria make the implantation of pacing and recording electrodes challenging. Consequently, rodent studies of atrial electrophysiology (EP) and AF substrate analysis are mainly done using either *ex vivo* preparations or invasive EP in deeply anesthetized rodents that are sacrificed at the end of the measurements.

Very little is known regarding the effects of RAP on the rodent atria. Until recently, only short-term RAP studies of several hours have been reported, mostly focusing on various molecular effects without actual data on AF substrate formation (Yamashita et al., 2000, 2003, 2005; Beharier et al., 2007; Yaegashi et al., 2016). Using a bi-atrial implantable device developed in our group, we previously managed to apply continuous RAP in unanesthetized rats under online confirmation of 1:1 atrial capture and serial measurements of AERP for up to 24 h (Etzion et al., 2008). Recently, a system based on a human leadless pacemaker was introduced for long-term single site pacing studies in rodents (Hulsmans et al., 2018). While this elegant and technically-advanced system demonstrated remarkable long-term pacing capabilities, the pacer itself could not be used for EP evaluation which was therefore done conventionally as a terminal invasive procedure under deep anesthesia (Hulsmans et al., 2018).

In the present study, we utilized our implantable rodent device to study the effect of sustained RAP on the AF substrate of unanesthetized rats and mice. Over time, continuous RAP invariably led to increased capture threshold and eventually to loss of capture in our system. Nevertheless, in both rats and mice constant RAP could be maintained for more than 7 consecutive days in some cases and daily assessment of AF substrate could be obtained for the first time in conscious rodents exposed to RAP. Under these conditions increased AF substrate was consistently observed in rats following 2 days of RAP while such finding could not be recapitulated in mice. Microarray analysis of transcriptional changes in the left atrium of rats following RAP [70 ms cycle length (CL)] vs. control pacing (140 ms CL) with similar ventricular response rates have indicated important transcriptional similarities between ATR of rats and large mammals. Overall, our findings support the notion that high excitation rates in the atria of rats can promote ATR and AF substrate formation over time.

## Materials and Methods

### Animals

The study was carried out in strict accordance with the Guide for the Care and Use of Laboratory Animals of the National Institute of Health. All animal studies reported in this article were approved by the institutional ethics committee of Ben-Gurion University of the Negev, Israel. Adult male C57BL6 mice (25–30 gr) and Sprague-Dawley rats (250–320 gr) were obtained from Envigo Laboratories (Jesusalem, Israel). The animals were kept in under standardized conditions throughout the study, according to home office guidelines: 12:12 light:dark cycles at 20–24°C and 30–70% relative humidity. Animals were free-fed autoclaved rodent chow and had free access to reverse osmosis filtered water. At the end of all experiments animals were euthanized under deep anesthesia.

### Implantable Devices and Surgical Techniques

**FIGURE 1:**
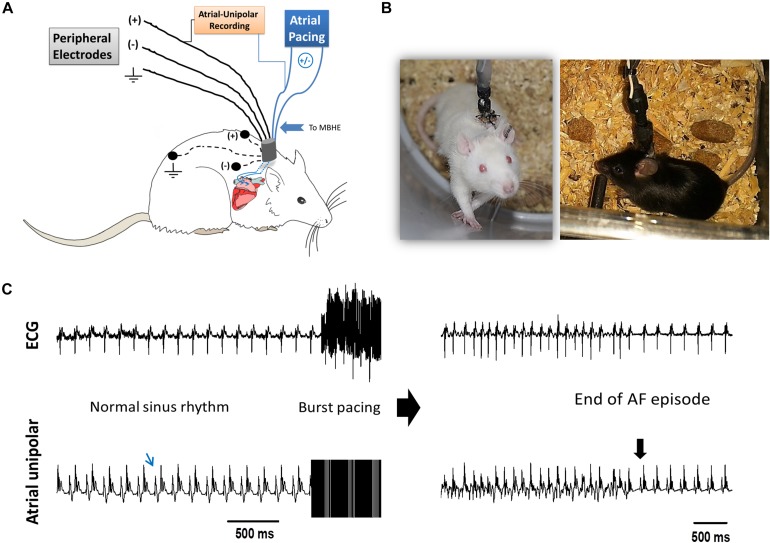
AF substrate analysis in unanesthetized rodents. **(A)** Schematic presentation of the experimental setup. MBHE was implanted on the RA for atrial pacing. One pole of the atrial electrode was also utilized to acquire high resolution recordings of the atrial signal (Atrial-Unipolar). **(B)** Photographs of an implanted rat and an implanted mouse freely moving in the EP cage during the RAP protocol. **(C)** Example of an AF episode triggered by burst pacing. This episode of 82 s was recorded from a rat that was exposed to RAP for 7 consecutive days (see Figure 2 for details). *Left:* Baseline ECG and atrial-unipolar recordings. *Middle:* Standard burst pacing protocol (20 s, 100 Hz, double threshold). *Right:* Post-burst recordings of an AF episode and its conversion to sinus rhythm. In each condition, 10 consecutive bursts were applied. AF substrate was evaluated in regard to the % of positive episodes (>1 s) and the total AF duration.

The implantable devices that we used to pace the right atria (RA) of unanesthetized rats and mice as well as the implantation technique, were previously described in detail by our group (Etzion et al., 2008; Mor et al., 2014; Mulla et al., 2017, 2018). Briefly, the implantable device is composed of an 8-pin “female” connector that is attached by highly flexible insulated electrical wires to a single miniature-bipolar hook electrode (MBHE). Three additional single lead electrodes are utilized for grounding and peripheral ECG measurements. Before implantation, the 8-pin “female” connector was covered with a latex sheath to prevent direct contact with the subcutaneous tissue during the operation. Electron beam radiation was applied for sterilization of the device before its use. For device implantation animals were anesthetized (IP ketamine/xylazine 75/5 and 120/10 mg kg^–1^ for rats and mice, respectively) and mechanically ventilated. Under sterile conditions and constant heating, the MBHE was implanted on the RA, the peripheral electrodes were positioned in the animal’s back and the 8-pin “female” connector was exteriorized through the skin (Figures 1A,B). Post-operative recovery and analgesia were performed as described previously (Mulla et al., 2018).

### Pacing and Recording Apparatus

Following a 6 days recovery period, each animal was placed in a dedicated recording chamber where the 8-pin connector on the back of the animal was attached to the pacing and recording apparatus through an elastic electrical cable. In rats, the proximal part of the elastic cable was connected to a multi-channel commutator (PLA-SL12C/SB, PLASTICS One Inc., CA, United States) enabling free rotational movements of the rat in the cage without effects on the electrical connections. In mice, rotations in the flexible cable were released manually twice a day. In each animal the two poles of the atrial MBHE were electrically connected to an optically isolated pacing unit (STG4002-16 mA, Multichannels, Reutlingen, Germany) and two of the peripheral electrodes were utilized for ECG recordings (Amplifier 1700, A-M systems, Carlsborg, WA, United States). In addition, differential recordings between one pole of the MBHE and one of the ECG leads was used to record an ECG signal in which the atrial activity could be seen with high resolution (Atrial unipolar, Figure 1 and Supplementary Figure S1). Signals were filtered (1–1000 Hz) and sampled to the PC at a digital sample rate of 2 KHz using an A/D converter (PCI-6024E, National Instruments, Austin, TX, United States). A LabView-based program (National Instruments, Austin, TX, United States) controlled data acquisition and electrical stimulation. Pacing was applied using 4 ms symmetrical bi-phasic pulses.

### Study Design

Overall, 30 rats and 24 mice were used for the study. RAP experiments were initiated using 20 rats and 14 mice. Four rats and five mice died during the initial procedure. Out of the sixteen surviving rats, nine were used for RAP experiments and five of them reached the cutoff of at least 4 consecutive days of RAP. Seven additional rats were used as shams and their AF substrate was evaluated daily of 6–8 days. One sham had high capture threshold during the basal testing and was excluded. The nine mice that were successfully implanted were used for RAP experiments and seven of them reached the cutoff of at least 4 consecutive days of RAP and were used for the final analysis. For the rat microarray study 10 additional rats were utilized as detailed in the relevant section below. Since the RAP experiments in mice were totally negative we did not perform sham studies in mice. However, in order to quantify the minimal CL maintaining 1:1 atrial capture in conscious mice, bi-atrial MBHE implantation procedure was attempted as we previously performed successfully in rats (Etzion et al., 2008). This procedure led to excessive mortality in mice and thus after 10 successive failures the experiment was aborted.

### Sustained RAP With Repeated AF Substrate Analyses in Implanted Rats and Mice

Following overnight adaptation to the EP cage, baseline AF substrate analysis was performed during the early daytime hours of the circadian cycle, when animals were inactive. Diastolic capture threshold was confirmed at a pacing CL of 150 and 100 ms in rats and mice, respectively (Mulla et al., 2018). Next, AF substrate evaluation was performed including 10 consecutive bursts (20 s, double diastolic threshold, 10 ms CL). Positive episodes were defined as >1 s. AF inducibility (percentage of positive episodes), as well as total AF duration were calculated and summarized. Arrhythmic episodes were identified by recordings demonstrating more than one atrial signal per QRS and atrial signal which was clearly different in morphology in comparison with the regular atrial signal during sinus rhythm (Figure 1C). We did not discriminate between regular and irregular atrial rhythms in the current analysis since the atrial unipolar signal which was not purely atrial did not allow such discrimination in a fully accurate manner. After baseline measurements, continuous RAP was initiated: In the rats, a pacing CL of 70 ms was selected since such pacing was found to invariably maintain 1:1 atrial-capture in the unanesthetized state (Etzion et al., 2008). In mice, previous data on 1:1 atrial-capture was obtained only in the anesthetized state. Thus, a 50 ms CL was empirically selected. Atrial capture threshold was evaluated twice daily, and pacing intensity was adjusted accordingly. Every 24 h RAP was transiently stopped and AF substrate was re-evaluated until the atrial capture was eventually lost.

### Microarray Analysis of Rat Left Atrial Tissue Following RAP

For the microarray experiment all animals were treated with atenolol (0.5 mg/ml) in the drinking water in order to reduce AV conduction during RAP, as previously described (Etzion et al., 2008). Following an overnight adjustment period in the pacing cage the rats were subjected to either RAP protocol (70 ms CL) or control pacing treatment (140 ms CL). The ventricular response rate during RAP at 70 ms CL was invariably 2:1, resulting in a RR interval of 140 ms. In contrast, the ventricular response rate during control pacing at 140 ms CL was invariably 1:1. Thus, in both groups pacing led to an identical ventricular response rate leaving the atrial excitation rate as the sole differential parameter (Figure 4A). Following 2 days of pacing the animals were injected with heparin (500 mg/kg, IP) and 10 min later were sacrificed under deep anesthesia. The hearts were gently rinsed with phosphate buffered saline to remove the blood cells and the left atria were excised, snap-frozen in liquid nitrogen and stored at –80°C. In order to confirm uniform conditions each experiment was conducted on a pair on rats operated and recovered under similar conditions and exposed to RAP or control pacing in adjacent cages at the same time.

RNA samples were extracted from the left atrium with TRI-Reagent (MRC, Cincinnati, OH, United States). The T-RNA samples were analyzed for concentration and purity using the NanoDrop ND-100 Spectrophotometer (NanoDrop Technologies, Wilmington, DE, United States). All RNAs displayed a 260/280 optical density ratio > 1.9. RNA integrity was assessed by an Agilent 2100 Bioanalyzer (Agilent Technologies, Palo Alto, CA, United States). All RNAs displayed a RNA Integrity Number (RIN) > 8. Microarray hybridization was performed at the DNA microarray laboratory core facility, National Institute for Biotechnology in the Negev (NIBN), using the “GeneChip^®^ Whole Transcript (WT) Sense Target Labeling Assay” protocol of Affymetrix (Santa Clara, CA, United States). Briefly, 100 ng total RNA from each sample was used as input to synthesize an adequate amount of ss-cDNA which in turn was fragmented and biotinylated. The biotinylated samples were hybridized to the GeneChip Rat Gene 1.0 ST arrays containing 722,254 probe sets (for an estimated 27,342 genes). Following hybridization the arrays were washed and stained using FS450_0007 protocol, and fluorescent signals were scanned using the 3000 G7 Scanner (Affymetrix).

### Microarray Data Analysis

Microarray CEL files were processed in Partek Genomics Suite^®^ using Robust Multiarray Averaging (RMA, Irizarry et al., 2003) with default parameters. Signal distribution plots, principal component analysis and hierarchical clustering of the normalized samples were examined, in order to assess the quality of the microarrays. The principal component analysis clarified matching between the original pairs of RAP and control pacing rats. Statistical hypothesis testing for identification of differentially expressed genes between RAP and control pacing samples was performed using paired student *t*-test. Differentially expressed genes were defined as those having absolute expression signal (log2) > 5 in at least one of the arrays, *p*-value < 0.05, and fold of change > 1.3 (in linear scale) in either direction. The expression data were deposited at the Gene Expression Omnibus (accession: GSE126711). Enrichment analysis was performed with MetaCore^TM^ (Thomson Reuters).

### Validation RT q-PCR Measurements

Microarray analysis was validated by the expression pattern of selected up-regulated and down-regulated genes using reverse transcriptase-quantitative polymerase chain reaction (RT-qPCR) experiments (Real Time PCR System Instrument – 7300, Applied Biosystems) with PerfeCTa SYBR Green FastMix (Quanta BioSciences). The selected genes were chosen based on a fold change >1.5 and possible interest in their role in ATR based on existing literature. Synthesis of cDNA for RT-qPCR was performed using random hexamers and Taqman reverse transcription reagents according to the manufacturer’s protocol (Applied Biosystems). Primers for target genes were designed by Biosearch Technology and synthesized by Agentek, Israel (Supplementary Table S1). For each gene, atrial mRNA from RAP and control pacing treated rats were analyzed. Cycling conditions were: 95°C for 10 min, followed by 35 cycles of 95°C for 30 s, 60°C for 15 s and 72°C for 30 s, and a final melting step (78–99°C). The calculation of relative change in mRNA was performed with the efficiency 2^–ΔΔ*CT*^ method, with the expression of the genes of interest normalized to Hypoxanthine Phosphoribosyltransferase 1 (HPRT1) gene.

### LA MicroRNA Expression

The expression of specific MicroRNAs (miRNAs) including miR-1, miR-26, miR-101, and miR-328 was evaluated. For detection of miRNAs in samples, cDNA was synthesized using the qScript microRNA cDNA synthesis kit according to the manufacturer’s instruction (Quanta Biosciences). RT-qPCR miRNA assay was performed using the PerfeCTa SYBR Green SuperMix (Quanta Biosciences) with specific primers (Supplementary Table S2). The small RNA molecule U6 small nuclear (HS-RNU6) was amplified as a control.

### Statistical Analysis

In each group of animals (RAP and control pacing) AF substrate parameters in the first 2 days were compared to the AF substrate parameters in the following days (until capture was lost) using paired non-parametric testing (Wilcoxon). Comparison between the paced and sham groups was done using unpaired non-parametric testing (Mann–Whitney). RT-qPCR results were compared to the microarray data using linear regression analysis. The level of significance was set at *p* ≤ 0.05. Microarray statistics and analysis are described separately above.

## Results

### RAP Promotes AF Substrate Formation in the Rat

**FIGURE 2:**
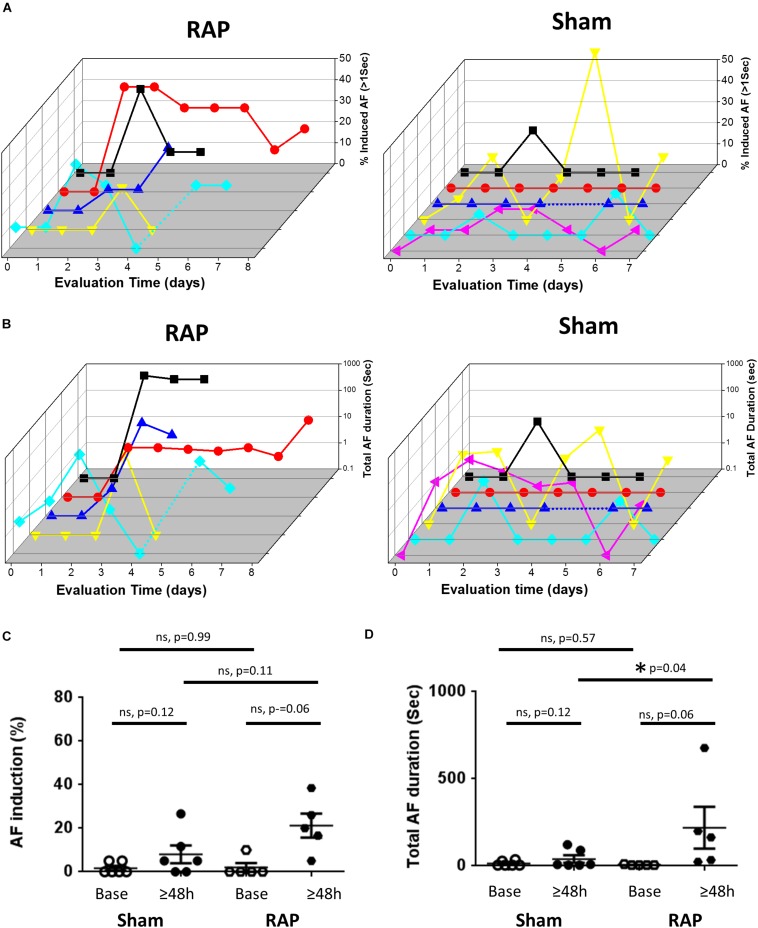
Increased AF substrate in rats exposed to RAP. **(A)** Analysis of AF induction. 3D plots of daily evaluation in five RAP rats (left) and six Sham rats (right). In both plots each color represents a different rat. For the RAP animals evaluation was continued until capture was lost or was too high to keep with the RAP. For the Shams, evaluation was maintained for 6–8 days. Note absence of AF induction in the majority of rats at baseline as well as following 1 day of RAP. Also note increased AF substrate following 2 days of RAP. **(B)** Analysis of Total AF duration. 3D representation as in **(A)** above. **(C,D)** Comparison of the AF substrate parameters before and after 2 days in the RAP vs. Sham rats. Note the strong tendency of increase in AF induction and AF duration following 2 days in the RAP group. Also note increased AF duration following 2 days in the RAP group vs. the Sham group.

Since the AF substrate of conscious rodents was never evaluated previously, we first examined the basal substrate in unanesthetized rats and mice under baseline conditions, i.e., following 6 days of recovery from implantation surgery and an additional day of acclimation in the EP cage. Thereafter, continuous RAP was initiated and AF substrate was evaluated on a daily basis. In both species, we found very low AF substrate under basal conditions. However, in five rats in which RAP was maintained for at least 4 consecutive days, AF episodes consistently developed following 2 days (Figures 2A,B). Statistical comparison between the average AF substrate parameters before 2 days of RAP vs. following 2 days of RAP indicated a strong tendency of increase in both AF inducibility and duration (Figures 2C,D, *p* = 0.06, for both). Such a finding that characterized the rats exposed to RAP was not observed in six additional rats which were evaluated for 6–8 days under exactly similar conditions apart from the RAP (sham group). Indeed, statistical comparison between the sham and RAP groups indicated increased total AF duration in the RAP group after 2 days. AF inducibility also demonstrated a similar tendency of increase in the RAP group. However, it did not reach significance (Figures 2C,D).

**FIGURE 3:**
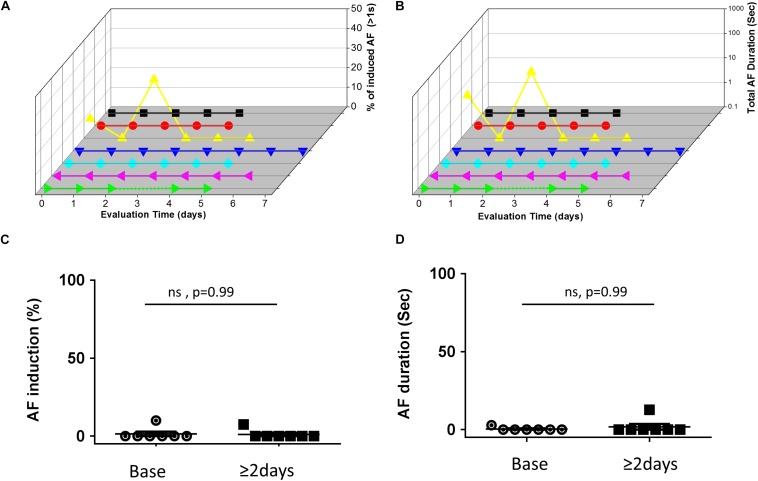
Absence of AF substrate augmentation in mice exposed to RAP. **(A,B)** Analysis of AF induction and AF duration in seven mice in which RAP could be maintained for up to 7 consecutive days. 3D plots of daily AF evaluation as in Figure 2. Note that only one animal demonstrated AF episodes and no clear effect of the RAP could be detected. **(C,D)** Summary analysis comparing the AF substrate before and after 48 h of continuous RAP at CL = 50 ms. Note absence of AF substrate augmentation following RAP in mice.

In contrast to the findings in rats, mice in which RAP was maintained for up to 7 consecutive days at a CL of 50 ms did not show any increase in the AF substrate parameters (Figure 3). In order to interpret the negative findings appropriately it was critical to know whether 1:1 capture was actually maintained by the RAP at this CL. Thus, we next aimed to perform a bi-atrial MBHE implantation procedure as we previously performed in rats (Etzion et al., 2008). Unfortunately, this procedure was associated with high mortality and was eventually aborted. Thus, assessment of the minimal CL maintaining 1:1 atrial capture in conscious mice could not be achieved.

### Transcriptional Remodeling in the Left Atrium of Rats Exposed to RAP

**FIGURE 4:**
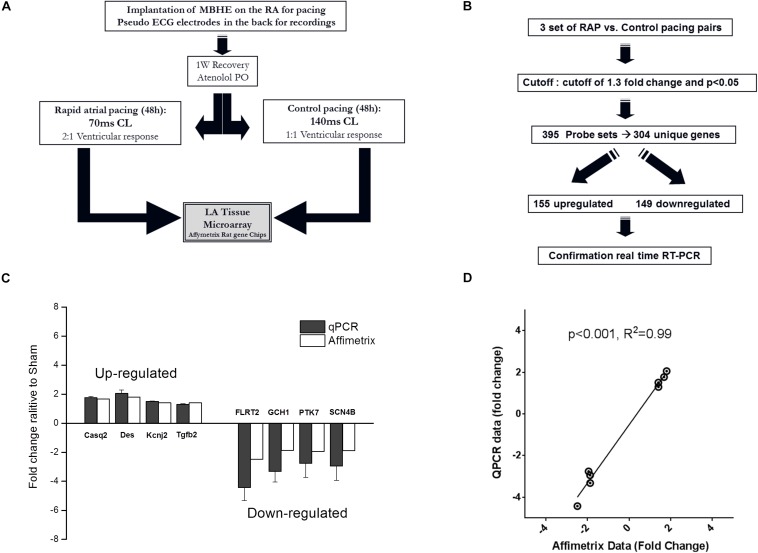
Paradigm for evaluation of transcriptional effects of RAP on left atrial tissue of rats. **(A,B)** Schematic diagrams of microarray study design and basic outcome. **(C,D)** RT-qPCR for eight genes of interest indicating data reliability.

To further characterize the effects of RAP on the rat atria we performed microarray analysis and determined the differential effects of RAP vs. near normal pacing for 2 days on the gene expression profile of the left atrium (Figure 4A). Five initial pairs of RAP vs. control paced rats successfully terminated the experimental protocol, each pair in two adjacent cages at the same time. However, high quality RNA samples were obtained only for three of these pairs which were further used for the microarray analysis. Principle component analysis demonstrated clear similarities within each pair (not shown). Using a cutoff of 1.3-fold change and *p* < 0.05 we identified 395 sets of probes that were differentially expressed in the RAP vs. control paced rats. Following extraction of unidentified probesets and correction for duplicate gene symbols our final analysis included 304 differential genes; 155 upregulated and 149 downregulated (Figure 4B). RT-qPCR for eight genes of interest (four upregulated and four downregulated) confirmed excellent linear correlation with the affymetrix data (Figures 4C,D). A complete list of significantly regulated transcripts is provided in Supplementary Table S3.

Significantly regulated genes were classified and analyzed with MetaCore^TM^ pathway analysis software in which differentially expressed genes are attributed to pathway maps and process networks. Table 1 indicates the maps and networks significantly affected by RAP in rats (*p* < 0.05). Differentially regulated maps and networks revealed functional cross-link between RAP and genes predominantly in functional classes that include development, inflammation, apoptosis, as well as those involved in the immune response to stress. Specifically, epithelial-to-mesenchymal transition (EMT), Transforming growth factor-β (TGF-β) signaling and IL-6 signaling were significantly affected by RAP (Table 1). These findings indicate clear relevance of the effects of RAP to the atrial remodeling of human subjects with AF (see section Discussion).

**TABLE 1:** Enrichment analysis of LA gene expression in response to RAP.

**No.**	**Pathway maps**	***p*-value**	**Ratio**
1	Development_TGF-beta-dependent induction of EMT via MAPK	2.112E-03	4/47
2	Immune response_Lectin induced complement pathway	2.465E-03	4/49
3	Cytoskeleton remodeling_Neurofilaments	2.936E-03	3/25
4	Immune response_Classical complement pathway	3.067E-03	4/52
5	Immune response_IL-6 signaling pathway	4.510E-03	3/29
6	Mucin expression in CF via IL-6, IL-17 signaling pathways	7.083E-03	3/34
7	Immune response_Oncostatin M signaling via MAPK in mouse cells	7.682E-03	3/35
8	Development_TGF-beta-dependent induction of EMT via SMADs	7.682E-03	3/35
	Immune response_Oncostatin M signaling via MAPK in human cells	8.968E-03	3/37
10	Immune response_Human NKG2D signaling	9.656E-03	3/38

**No.**	**Process networks**	***p*-value**	**Ratio**

1	Development_Skeletal muscle development	1.561E-03	8/144
2	Muscle contraction	4.866E-03	8/173
3	Inflammation_IL-6 signaling	9.594E-03	6/119
4	Apoptosis_Apoptotic nucleus	1.076E-02	7/159
5	Development_Regulation of angiogenesis	2.060E-02	8/223
6	Inflammation_Complement system	2.519E-02	4/73
7	Inflammation_Amphoterin signaling	3.426E-02	5/118
8	Cytoskeleton_Intermediate filaments	3.512E-02	4/81

**Genes showing changes in expression after 48 h of rapid atrial pacing (70 ms CL) vs. control pacing (140 ms CL) were detected using Affymetrix Rat genome array and were analyzed with MetaCore pathway analysis. Pathway Maps: The canonical pathways used in this analysis represent a set of ∼650 maps generated from the GeneGo database (GeneGo, St. Joseph, MI, United States) covering human biology (signaling and metabolism) in a comprehensive way. Process Networks: about 110 cellular and molecular processes whose content is defined and annotated by GeneGo. Each process represents a pre-set network of protein interactions characteristic for the process. The table indicates the top maps and networks affected by ATR in the rat. Ratio indicates the number of differentially expressed genes relative to the total number of genes in each map or network.**

### Transcriptomic Signature in the LA of Rats Exposed to RAP Compared With Human AF

Previously reported microarray results by Mace et al. (2009), identified similarities with respect to large-scale patterns of transcriptional remodeling between rapidly stimulated HL-1 myocytes and human AF (Mace et al., 2009). To investigate whether the LA tissue of rats subjected to RAP demonstrates transcriptional similarities with human AF, the differentially regulated transcripts of our data set were compared to the data of Barth et al. (2005), which was utilized for the comparison with HL-1 cells by Mace et al. (2009). The pathway maps of this analysis represent the most dominantly regulated pathways in the rat RAP data set, which were also differentially regulated in the chronic human AF data. Interestingly, the first two pathways which came up in this analysis as well as three additional pathway maps are related to cellular metabolism and specifically to carbohydrate metabolism (Table 2). In human AF, the pattern of adaptation that occurs in metabolic transcripts resembles a fetal energy program (Barth et al., 2005; Kim et al., 2005) including up-regulation of several transcriptions of glycolytic enzymes, suggesting a switch to glucose utilization from fatty acid oxidation. Thus, the results of this analysis indicate that RAP in the rat can lead to transcriptional changes which may resemble human AF particularly in respect to metabolism.

**TABLE 2:** Enrichment analysis: Rat RAP vs. Human AF.

**No.**	**Pathway maps**	***p*-value**	**Ratio**
1	Glycolysis and gluconeogenesis p. 1	2.231E-03	5/46
2	Glycolysis and gluconeogenesis (short map)	4.544E-03	8/66
3	Development_Ligand-dependent activation of the ESR1/AP-1 pathway	2.161E-02	6/14
4	Regulation of lipid metabolism_Regulation of acetyl-CoA carboxylase 2 activity in muscle	2.922E-02	3/19
5	Apoptosis and survival_NO signaling in apoptosis	3.527E-02	4/23
6	Transcription_Transcription regulation of aminoacid metabolism	3.828E-02	8/25
7	Muscle contraction_nNOS Signaling in Skeletal Muscle	4.279E-02	5/28
8	Histamine metabolism	4.428E-02	5/29
9	Transcription_Ligand-dependent activation of the ESR1/SP pathway	4.578E-02	4/30
10	Glycolysis and gluconeogenesis p. 2	4.876E-02	3/32

**Comparison between rat ATR and data set obtained for human AF (3). A comparison between the two gene lists was performed using the “compare experiments workflow” within MetaCore. 44 common genes (shared between the two input gene lists) were found. GeneGo Pathway Maps are as in table-1. The table indicates the 10 maps most commonly associated between the rat ATR and the human AF sets of data. Ratio indicates the number of differentially expressed genes in the Rat ATR set relative to the total number of genes in each map.**

**FIGURE 5:**
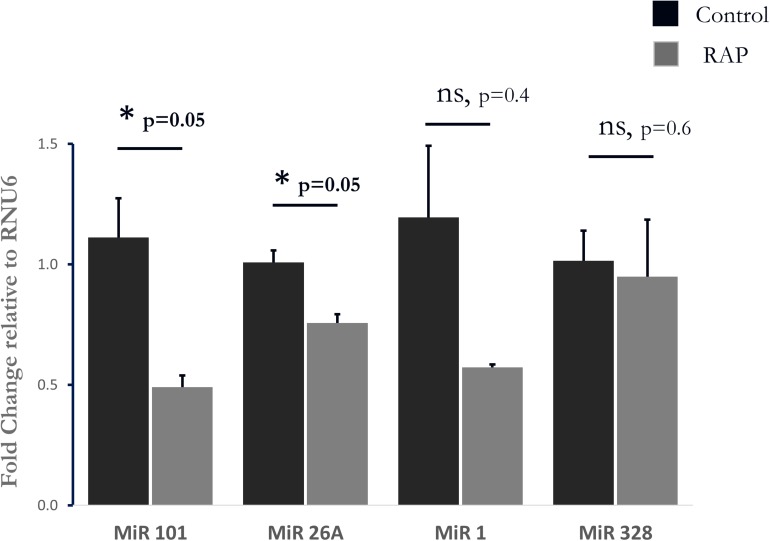
Analysis of four Micro RNAs known to be involved in AF-related remodeling. Note significant downregulation of MIR-101, and MIR-26A as well as a non-significant tendency of reduction in MIR-1. MIR-328 was unaffected by RAP (*n* = 3 for both RAP and control pacing, Mahan–Whitney test).

To further test the relevance of our results to those observed in human AF, we further investigated the transcriptional similarities at the level of individual transcripts. This comparison identified 19 out of 44 common transcripts that were regulated in a concordant manner in the rats subjected to RAP and in the human AF atrial samples (Table 3). An important finding in this context is the upregulation of KCNJ2 gene which encodes the Kir2.1 channel, which underlies the inward rectifier potassium current, IK1. Upregulation of Kir2.1 is a typical finding in ATR of large mammal models and gain-of-function mutation in this gene underlies a familial form of AF (Xia et al., 2005). Kir2.1 is regulated by NFAT signaling, which is tightly regulated through the calcineurin/NFAT signaling pathway and seems to play an important role in AF-related remodeling (Wakili et al., 2011; Harada et al., 2014). In this regard, the downregulation of FOXP1 (Table 3) is also in line with NFAT activation since this transcription factor is a known repressor of NFAT signaling in cardiomyocytes (Bai and Kerppola, 2011). Moreover, KCNJ2 upregulation has been linked to an inhibitory effect of NFAT activation on MIR-26 and possibly MIR-101 and MIR-1 (Wakili et al., 2011; Luo et al., 2013). Consistent with this mechanism we found significant down regulation of MIR-26 and MIR-101 and a trend of down regulation for MIR-1 in the rat LA samples subjected to RAP (Figure 5). Of note, while prominent upregulation of MIR-328 was also noted in AF-related remodeling (Lu et al., 2010) and was shown to inhibit the expression of CACNA1C and CACNB1, which encode cardiac L-type Ca^2+^ channel α1c- and β1 subunits, differential regulation of this MIR or the relevant subunits of the L-type Ca^2+^ channel were not observed in our RAP data set of the rat (Figure 5).

**TABLE 3:** Rat ATR vs. Human AF; commonly regulated genes.

**Common regulation**	**Gene symbol**	**Gene title**	**Metacore pathway/GO**
Up	AARSD1	Alanyl-tRNA synthetase domain containing 1	Alanyl-tRNA aminoacylation
	ADSL	Adenylosuccinate lyase	AMP biosynthetic process
	ASPN	Asporin	Negative regulation of transforming growth factor
	COQ6	Coenzyme Q6 homolog (*S. cerevisiae*)	Ubiquinone biosynthesis process
	FASTKD5	FAST kinase domains 5	Mitochondrion
	GAPDH	Glyceraldehyde-3-phosphate dehydrogenase	Glycolysis_and_ Gluconeogenesis
	KCNJ2	Potassium inwardly-rectifying channel, subfamily J, member 2	Ion transport
	MAFK	V-maf musculoaponeurotic fibrosarcoma oncogene homolog K (avian	Transcription, DNA-dependent
	PFKP	Phosphofructokinase, platelet	Glycolysis and Gluconeogenesis
	PPID	Peptidylprolyl isomerase D (cyclophilin D)	Protein folding
Down	Tcfe2a	Transcription factor E2a	Muscle cell differentiation/positive regulation of B cell proliferation
	FLRT2	Fibronectin leucine rich transmembrane protein 2	Cell adhesion
	FOXP1	Forkhead box P1	Specific transcriptional repressor activity
	OS9	Osteosarcoma amplified 9, endoplasmic reticulum lectin	Response to endoplasmic reticulum stress
	PON2	Paraoxonase 2	Response to oxidative stress
	TMEM108	Transmembrane protein 108	?
	CRTC3	CREB regulated transcription coactivator 3	Positive regulation of CREB transcription factor
	TRIM8	Tripartite motif-containing 8	Protein ubiquitination
	ZMIZ1	Zinc finger, MIZ-type containing 1	Regulation of transcription, DNA-dependent

*Comparison between rat ATR and a data set obtained for human AF (3) (Barth et al., 2005). The table indicates 19 genes that were regulated in a similar manner in both data sets.*

## Discussion

Sustained atrial tachycardia can modify the atrial properties of the large mammalian heart so that AF episodes recur and are maintained more readily over time (Wijffels et al., 1995; Nattel, 1999; Members et al., 2006). This ATR process is suggested to contribute to a variety of clinically important phenomena including the tendency of paroxysmal AF to become persistent, the tendency of AF to recur soon after cardioversion and the refractoriness of long-lasting AF episodes to pharmacological attempts of cardioversion. Experimentally, long-term RAP can stimulate ATR and eventually lead to sustained AF in large animal models (Wijffels et al., 1995; Yue et al., 1997). However, the effects of RAP in small mammalian models are far from being elucidated.

In this study, utilizing our unique experimental apparatus for EP studies in unanesthetized rodents (Mulla et al., 2017, 2018), we focused on the gap of knowledge in regard to ATR and AF substrate formation in rodents exposed to RAP. Although the duration of RAP was technically limited to several days in our system (see Limitations section), we could still obtain crucial experimental findings. Our results indicate that sustained RAP over several days increases the AF substrate of rats and recapitulates important molecular signatures of ATR that were previously described in large mammals and humans with AF. In contrast to the experiments in rats, which demonstrated increased AF substrate following more than 2 days of RAP, we did not find any indications for increased AF substrate formation in unanesthetized mice exposed to RAP for up to 7 consecutive days at 1200 bpm. These findings seem consistent with the recently reported results of Hulsmans et al. (2018). While Hulsmans et al. (2018) managed to perform long-lasting LA pacing using a leadless Medtronic pacemaker adapted for mice experimentation, AF inducibility tested in the anesthetized state at the end of 28 days demonstrated only a mild tendency of increase, which did not reach statistical significance. Interestingly, Hulsmans et al. (2018) also report molecular findings which are consistent with increased inflammatory response in the atrial tissue and are in line with our current microarray findings in the rats (discussed below). Thus, it is possible that although ATR in mice can promote AF substrate over time, this tendency is harder to detect due to the substantially lower basal AF substrate of mouse relative to the rat. Another option which should be taken into consideration is that atrial pacing at a rate of 1200 bpm (50 ms CL) does not lead to an actual 1:1 response in the atria of unanesthetized mice. Indeed, our previous findings in ICR mice under isoflurane anesthesia indicate that failure of 1:1 atrial capture may often occur in rates lower than 1200 bpm (Etzion et al., 2008). Thus, it is possible that the empirical RAP at 1200 bpm in the current study as well as in Hulsmans et al. (2018) did not actually lead to similar activation rate in the atria itself. Although we actively attempted to answer this question in the present study by implanting MBHEs on both atria, this experimental attempt technically failed. Thus, the answer for this critical question will have to wait for future technical advances.

### Transcriptional Analysis

Transcriptome analysis is among the most utilized approaches to study human disease at the molecular level (Casamassimi et al., 2017). Transcriptome analysis has proved to be a powerful approach to identify gene expression changes in response to AF and ATR (Barth et al., 2005; Kim et al., 2005; Mace et al., 2009; Deshmukh et al., 2015). Thus, we decided to utilize this approach to gain more insights into the ATR of rodents in the present study as well. Since such analysis is known to be highly sensitive to experimental confounders we had to carefully consider our experimental design. In this regard, our ability to create a model in which ventricular response is totally similar between RAP and control animals (Figure 4A) as well as the use of pairs of animals in which the implantation, recovery and actual pacing was done simultaneously, were all critical for the success of this approach.

The obtained results indicate that RAP in the rat induces atrial changes which may have high relevance for the pathophysiology of AF. For instance, EMT is an essential process during development, in which epithelial cells lose their epithelial markers and start to express fibroblast markers. The process of EMT also plays an important role in various pathological conditions, such as organ fibrosis and tissue repair (Kalluri and Neilson, 2003; Kalluri and Weinberg, 2009). Transforming growth factor-β (TGF-β) signaling which was dominantly affected according to our analysis, plays a central role in activation of fibrosis and is also an inducer of EMT. Inducible cardiomyocyte-specific deletion of Tgfbr2 in mice was shown to reduce fibrosis and improved cardiac function (Koitabashi et al., 2011). In contrast, cardiac overexpression of constitutively active TGF-β1 in mice resulted in selective atrial fibrosis, electrical conduction heterogeneity and AF susceptibility (Verheule et al., 2004).

The IL-6 signaling pathway, which was also found to be differentially regulated by RAP (Table 1; pathway map #5, Process network #3), has various pro inflammatory effects including stimulation of the synthesis of several acute-phase reaction proteins, such as fibrinogen and CRP, and endothelial cell activation and damage (Guo et al., 2012). Interestingly, several studies have indicated that elevated serum levels of IL-6 are associated with AF occurrence (Conway et al., 2004; Psychari et al., 2005; Marcus et al., 2010). IL-6 levels have also been positively correlated, not only with the presence of AF but also with its duration and with enlargement of left atrial diameter (Psychari et al., 2005). Additionally, prospective studies showed that high IL-6 levels independently predicted stroke in patients with AF (Lip et al., 2007; Roldan et al., 2012), and the addition of IL-6 resulted in improved prediction performance of the CHA2DS2-VASc clinical risk stratification schemes (Roldan et al., 2012). Complement activation (Table 1; pathway map #2, Process network #6) has also been described in patients with AF without other associated inflammatory diseases (Bruins et al., 1997). On the single gene level, the downregulation of gch1 which was also confirmed by the qPCR analysis (Figure 4C) is an intriguing finding. Gch1 is the first and rate-limiting enzyme in the *de novo* biosynthesis of tetrahydrobiopterin (BH4), an essential co-factor for all 3 isoforms of nitric oxide synthase (NOS) (Werner et al., 2011). NO is synthesized from L-arginine and oxygen by NOS using BH4 as a cofactor. Under conditions of low BH4 bioavailability, NOS is uncoupled to produce superoxide anion (O2–) instead of NO (Alkaitis and Crabtree, 2012). A growing body of evidence suggests that NOS uncoupling is involved in atrial remodeling and the pathogenesis of AF (Schotten et al., 2011; Bonilla et al., 2012). The comparison of rat RAP findings with human AF data support similarities in metabolic changes and demonstrate indirect but compelling evidence for the activation of NFAT signaling in the rat RAP samples as has been demonstrated in large mammalian models of ATR (Wakili et al., 2011).

At this point it is hard to determine which of the transcriptional changes observed in the present study may be linked to the increased AF substrate that we identified following 48 h of RAP. It seems unlikely that TGF-^β^ signaling leading to fibrosis may have such a rapid effect on AF substrate formation. While KCNK2 upregulation leading to AERP shortening may be a plausible mechanism, our previous findings in rats as well as the recent findings of Hulsmans et al. (2018) in mice (Hulsmans et al., 2018) do not support persistent AERP shortening in rodents exposed to RAP. Of note, while absence of AERP shortening may be regarded as a clear disadvantage of the rodent RAP model in mimicking AF-related remodeling, our current data may suggest that this model may be further utilized to access the effects of RAP which can enhance AF substrate formation regardless of AERP shortening. Such mechanisms are clearly harder to access in the classical large mammalian models in which AERP shortening is among the most dominant findings.

### Limitations

The inability to maintain RAP for more than several days with our MBHE is a clear drawback of our system. This limitation is clearly related to the application of continuous pacing through the electrode which invariably leads to increase of the capture threshold over time (Supplementary Figure S2) and is not detected if pacing is not applied through the electrode. Nevertheless, while this study was designed and operated our MBHE was still the only published methodology for implantation of atrial pacing electrodes in rodents (Etzion et al., 2008; Mulla et al., 2017, 2018). Certainly, the recent proof of concept of Hulsmans et al. (2018) demonstrating far more stable pacing capabilities urge for improvements in our system as well. Nevertheless, the ability of our system to be utilized for repeated EP evaluations of rodents in the conscious state is currently unique and was a crucial component of the current study. A clear additional limitation of the current setup was the inability to obtain additional EP parameters such as AERP and conduction velocity. This is related to the fact that only two electrical poles were implanted on the RA in the current MBHE design and such setup does not allow clear detection of the atrial signal during pacing (Etzion et al., 2008). Clearly, a future improvement of the system enabling the insertion of two pairs of electrical poles (one pair for pacing and additional one for recording) would be of great value. The short duration of arrhythmic episodes induced by burst pacing in rodents is another limitation that should be considered. However, it has been shown in several studies that the susceptibility to this form of arrhythmia is affected by multiple etiological factors of clinical relevance to human AF (Iwasaki et al., 2014; Hohl et al., 2017; Lau et al., 2017).

Finally, false discovery rate (FDR) was not used for analysis of the microarray data, since the signals we detected were not strong enough to pass the FDR cutoff. Nevertheless, it should be kept in mind that the methodology we used in order to confirm similar ventricular response rate and confine our findings to the effect of RAP only, restricted the biological differences in atrial response rate between the RAP and the control animals (as it induced certain degree of atrial tachypacing in the control animals as well). In addition, our microarray findings were clearly validated using real-time RT PCR experiments. Thus, regardless of this limitation the findings are highly specific to RAP and are validated in biological terms.

## Data Availability Statement

The datasets generated for this study can be found in GEO, GSE126711.

## Ethics Statement

The study was carried out in strict accordance with the Guide for the Care and Use of Laboratory Animals of the National Institutes of Health. All animal studies reported in this article were approved by the institutional ethics committee of Ben-Gurion University of the Negev, Israel.

## Author Contributions

WM, BH, SEl, MMo, MMu, and HK-G performed the experiments. WM, IP, and SEt analyzed the data and interpreted the results of the experiments. WM, SEt, and YE prepared the figures. WM and YE drafted the manuscript. RG, MMu, HK-G, and VC-C edited and revised the manuscript. All authors approved the final version of the manuscript.

## Conflict of Interest

The authors declare that the research was conducted in the absence of any commercial or financial relationships that could be construed as a potential conflict of interest.
