# Supplementary material for: Rapid Atrial Pacing Promotes Atrial Fibrillation Substrate in Unanesthetized Instrumented Rats
Source: Front Physiol. 2019 Sep 20;10:1218. doi: 10.3389/fphys.2019.01218 (PMC6763969; doi:10.3389/fphys.2019.01218)
Supplement: Supplementary file 1 [file Image_1.pdf]

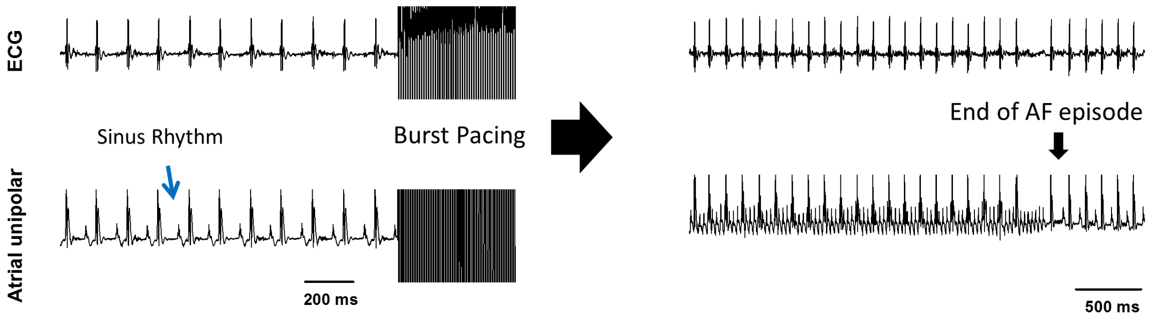


**Figure S1: Example of an atrial arrhythmic episode triggered by burst pacing in a conscious C57Bl/6 mice.** *Left:* Baseline ECG and atrial-unipolar recordings. *Middle:* Standard burst pacing protocol (20 seconds, 100 Hz, double threshold). *Right:* Post-burst recordings of a regular arrhythmic episode and its conversion to sinus rhythm (arrow).
