# Supplementary material for: Rapid Atrial Pacing Promotes Atrial Fibrillation Substrate in Unanesthetized Instrumented Rats
Source: Front Physiol. 2019 Sep 20;10:1218. doi: 10.3389/fphys.2019.01218 (PMC6763969; doi:10.3389/fphys.2019.01218)
Supplement: Supplementary file 2 [file Image_2.pdf]

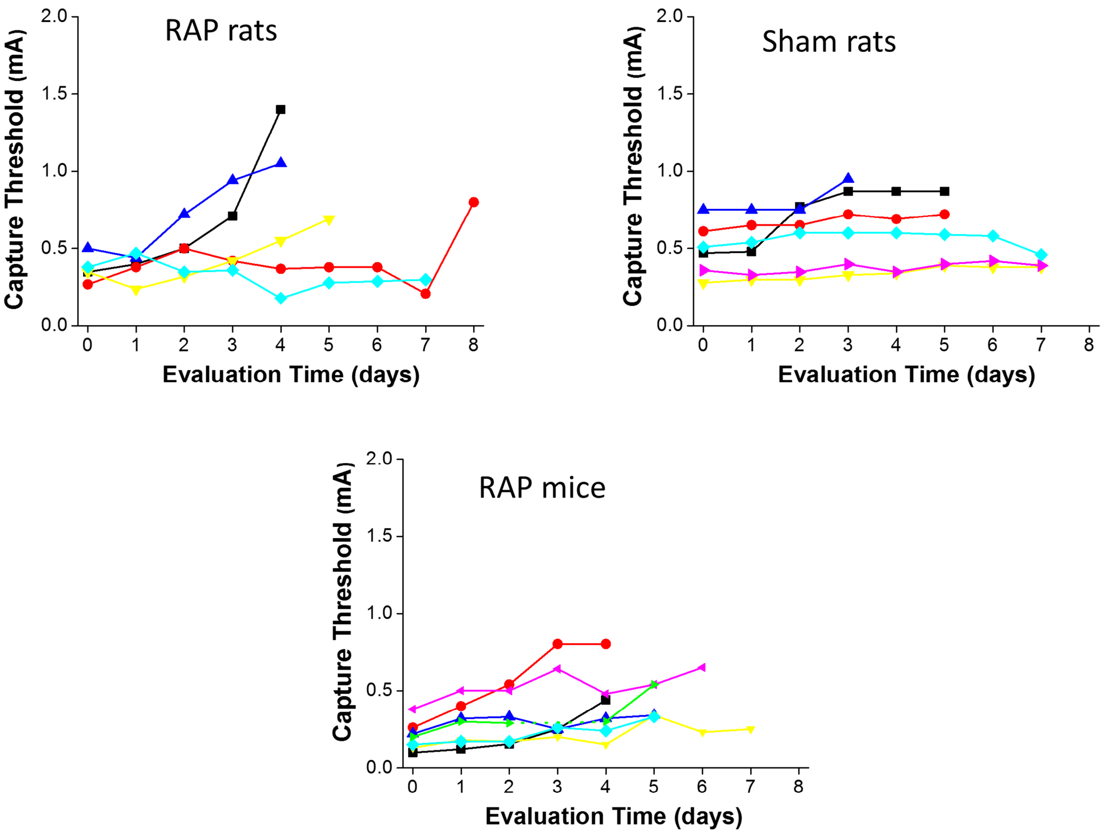

Figure S2: Daily trial capture threshold measurements of conscious rats and mice. Each plot represents an animal recorded for at least four consecutive days in the EP apparatus. For rats see AF substrate analysis results in Figure 2. Colors for each animal are matched with Figure 2. For mice see AF substrate analysis results in Figure 3.
