# Supplementary material for: Rapid Atrial Pacing Promotes Atrial Fibrillation Substrate in Unanesthetized Instrumented Rats
Source: Front Physiol. 2019 Sep 20;10:1218. doi: 10.3389/fphys.2019.01218 (PMC6763969; doi:10.3389/fphys.2019.01218)
Supplement: Supplementary file 3 [file Table_1.docx]

**Mulla et al., Front. Physiol. | doi: 10.3389/fphys.2019.01218**

**Table S1:** Rat primers for genes of interest.

| Reverse primer | Forward primer |  |  |
| --- | --- | --- | --- |
| TTCCACCAAGACATCTGCTGC | CCAAGAGGCTGGGCTTCA | NM_017131.2 | **Casq2** |
| CAGTTGGATTTCCTCCTGTAGCTT | CCGAGCTCTACGAGGAGGAGAT | NM_022531.1 | **Des** |
| TCGCCTGGTTGTGGAGATCT | TGAAAATGAAGTTGCCCTAACAAG | NM_017296.1 | **Kcnj2** |
| TTGAAGCATTCTTCTCCATTGTTG | GTGCTCTGTGGGTACCTTGATG | NM_031131.1 | **Tgfb2** |
| GATGGCCACTGCAATCTGTTT | GCAAGTCCTTGGTCTCAGCAA | NM_024356 | **Gch1** |
| AGCTTCTTCAGCAACAGGATGC | TTCCTCCAAGTGGTTGATAAATTG | NM_001008880 | **Scn4b** |
| TGGCGGAAGTGGTAGTATTCACT | GGACTTAGCTGCTCGCAACTG | NM_001106889 | **Ptk7** |
| GCGTGATGCCAAGTACTGTCA | GCTAAGGAGACAAGGCTACCAGAT | NM_001106750 | **Flrt2** |
| GTAGATTCAACTTGCCGCTGTCT | GAGCACTTCAGGGATTTGAATCAT | NM_012583.2 | **Hprt1** |
