# Supplementary material for: Rapid Atrial Pacing Promotes Atrial Fibrillation Substrate in Unanesthetized Instrumented Rats
Source: Front Physiol. 2019 Sep 20;10:1218. doi: 10.3389/fphys.2019.01218 (PMC6763969; doi:10.3389/fphys.2019.01218)
Supplement: Supplementary file 4 [file Table_2.docx]

**Mulla et al., Front. Physiol. | doi: 10.3389/fphys.2019.01218**

**Table S2:** Rat primers for miRs of interest.

| HSMIR-0101 | UACAGUACUGUGAUAACUGAA |
| --- | --- |
| HSMIR-0026A | UUCAAGUAAUCCAGGAUAGGCU |
| RNMIR-0001 | UGGAAUGUAAAGAAGUGUGUAU |
| HSMIR-0328 | CUGGCCCUCUCUGCCCUUC |
| HS-RNU6 | GUGCCUGCUUCGGCAGCACAUAUACUAAAAUUGGAACGAUACAGAGAAGAUUAGCAUGGCCCCUGCGCAAGGAUGACACGCAAAUUCGUGAAGCGUUCCAUAUUUU |
