# Supplementary material for: Rapid Atrial Pacing Promotes Atrial Fibrillation Substrate in Unanesthetized Instrumented Rats
Source: Front Physiol. 2019 Sep 20;10:1218. doi: 10.3389/fphys.2019.01218 (PMC6763969; doi:10.3389/fphys.2019.01218)
Supplement: Supplementary file 5 [file Table_3.docx]

**Mulla et al., Front. Physiol. | doi: 10.3389/fphys.2019.01218**

**Table S3:** Significantly regulated genes in the LA of rats: RAP vs Control pacing (2 days).

Click on the table to see the full list of genes in an Excel format.
